# Benefits of VCE-003.2, a cannabigerol quinone derivative, against inflammation-driven neuronal deterioration in experimental Parkinson’s disease: possible involvement of different binding sites at the PPARγ receptor

**DOI:** 10.1186/s12974-018-1060-5

**Published:** 2018-01-16

**Authors:** Concepción García, María Gómez-Cañas, Sonia Burgaz, Belén Palomares, Yolanda Gómez-Gálvez, Cristina Palomo-Garo, Sara Campo, Joel Ferrer-Hernández, Carolina Pavicic, Carmen Navarrete, M. Luz Bellido, Moisés García-Arencibia, M. Ruth Pazos, Eduardo Muñoz, Javier Fernández-Ruiz

**Affiliations:** 1 Instituto Universitario de Investigación en Neuroquímica, Departamento de Bioquímica y Biología Molecular, Facultad de Medicina, Universidad Complutense, 28040 Madrid, Spain; 2 Centro de Investigación Biomédica en Red de Enfermedades Neurodegenerativas (CIBERNED), Madrid, Spain; 3 Instituto Ramón y Cajal de Investigación Sanitaria (IRYCIS), Madrid, Spain; 4 Instituto Maimónides de Investigación Biomédica de Córdoba (IMIBIC), Córdoba, Spain; 5 Departamento de Biología Celular, Fisiología e Inmunología, Universidad de Córdoba, Córdoba, Spain; 6 Hospital Universitario Reina Sofía, Córdoba, Spain; 7 Innohealth Group, Madrid, Spain; 8 Vivacell Biotechnology España, Córdoba, Spain; 9 Present address: Institute for Science and Technology in Medicine, Keele University, Newcastle under Lyme, UK; 10 Present address: Department of Biochemistry and Molecular Biology, Universidad de Las Palmas de Gran Canaria, Las Palmas, Spain; 11 Present address: Laboratorio de Apoyo a la Investigación. Hospital Universitario Fundación Alcorcón, Alcorcón, Madrid, Spain

**Keywords:** Cannabinoids, VCE-003.2, PPARγ receptors, Inflammation, Microglial activation, LPS, Parkinson’s disease

## Abstract

**Background:**

Neuroprotection with cannabinoids in Parkinson’s disease (PD) has been afforded predominantly with antioxidant or anti-inflammatory cannabinoids. In the present study, we investigated the anti-inflammatory and neuroprotective properties of VCE-003.2, a quinone derivative of the non-psychotrophic phytocannabinoid cannabigerol (CBG), which may derive its activity at the peroxisome proliferator-activated receptor-γ (PPARγ). The compound is also an antioxidant.

**Methods:**

We evaluated VCE-003.2 in an in vivo [mice subjected to unilateral intrastriatal injections of lipopolysaccharide (LPS)] model of PD, as well as in in vitro (LPS-exposed BV2 cells and M-213 cells treated with conditioned media generated from LPS-exposed BV2 cells) cellular models. The type of interaction of VCE-003.2 at the PPARγ receptor was furtherly investigated in bone marrow-derived human mesenchymal stem cells (MSCs) and sustained with transcriptional assays and in silico docking studies.

**Results:**

VCE-003.2 has no activity at the cannabinoid receptors, a fact that we confirmed in this study using competition studies. The administration of VCE-003.2 to LPS-lesioned mice attenuated the loss of tyrosine hydroxylase (TH)-containing nigrostriatal neurons and, in particular, the intense microgliosis provoked by LPS in the substantia nigra, measured by Iba-1/Cd68 immunostaining. The analysis by qPCR of proinflammatory mediators such as tumor necrosis factor-α (TNF-α), interleukin-1β (IL-1β), and inducible nitric oxide synthase (iNOS) in the striatum showed they were markedly elevated by the LPS lesion and strongly reduced by the treatment with VCE-003.2. The effects of VCE-003.2 in LPS-lesioned mice implied the activation of PPARγ receptors, as they were attenuated when VCE-003.2 was co-administered with the PPARγ inhibitor T0070907. We then moved to some in vitro approaches, first to confirm the anti-inflammatory profile of VCE-003.2 in cultured BV2 cells exposed to LPS. VCE-003.2 was able to attenuate the synthesis and release of TNF-α and IL-1β, as well as the induction of iNOS and cyclooxygenase-2 (COX-2) elicited by LPS in these cells. However, we found such effects were not reversed by GW9662, another classic PPARγ antagonist. Next, we investigated the neuroprotective effects of VCE-003.2 in cultured M-213 neuronal cells exposed to conditioned media generated from LPS-exposed cultured BV2 cells. VCE-003.2 reduced M-213 cell death, but again, such effects were not reversed by T0070907. Using docking analysis, we detected that VCE-003.2 binds both the canonical and the alternative binding sites in the PPARγ ligand-binding pocket (LBP). Functional assays further showed that T0070907 almost abolished PPARγ transcriptional activity induced by rosiglitazone (RGZ), but it did not affect the activity of VCE-003.2 in a Gal4-Luc system. However, T0070907 inhibited the effects of RGZ and VCE-003.2 on the expression of PPARγ-dependent genes upregulated in MSCs.

**Conclusions:**

We have demonstrated that VCE-003.2 is neuroprotective against inflammation-driven neuronal damage in an in vivo model of PD and in in vitro cellular models of neuroinflammation. Such effects might involve PPARγ receptors, although in silico and in vitro experiments strongly suggest that VCE-003.2 targets PPARγ by acting through two binding sites at the LBP, one that is sensitive to T0070907 (canonical binding site) and other that is not affected by this PPARγ antagonist (alternative binding site).

**Electronic supplementary material:**

The online version of this article (10.1186/s12974-018-1060-5) contains supplementary material, which is available to authorized users.

## Background

Inflammation is a key pathogenic event in Parkinson’s disease (PD), so that anti-inflammatory strategies are being investigated to limit neuronal deterioration in this disease [1]. Certain cannabinoids have proved important anti-inflammatory/neuroprotective properties, which have been primarily assigned to the role exerted by the cannabinoid receptor type-2 (CB_2_) in the control of glia-dependent inflammatory events typical of neurodegenerative/neuroinflammatory disorders [2, 3]. However, its relevance in PD has remained elusive for years. A few years ago, Price and coworkers [4] described an elevation of CB_2_ receptors in microglial cells recruited at the lesion sites in mice intoxicated with MPTP, a model with a modest glial response. These authors found that targeting these receptors reduced the damage of nigrostriatal neurons [4], although a further study showed that the inhibition of microglial activation and the preservation of nigrostriatal dopaminergic neurons in MPTP-lesioned mice involved surprisingly the activation of the cannabinoid receptor type-1 (CB_1_) too [5]. In our laboratory, we worked with postmortem basal ganglia collected from PD patients and confirmed such upregulatory response of CB_2_ receptors in glial elements [6]. We also investigated the issue in an inflammatory model of nigrostriatal damage consisting in intrastriatal injection of lipopolysaccharide (LPS), in which we found elevated levels of CB_2_ receptors in the basal ganglia [6, 7]. Such receptors may be apparently located in activated glial elements, although we did not investigate the cell substrates in which this response takes place. In addition, we found that CB_2_ receptor-deficient mice were more vulnerable to LPS lesion than wild-type animals [6, 7], a difference that was not found in a model with poor inflammatory responses, mice lesioned with 6-hydroxydopamine, in which the death of dopaminergic neurons is related to mitochondrial dysfunction and oxidative damage [7]. In agreement with this difference, LPS-lesioned mice responded to compounds targeting the CB_2_ receptor by preserving tyrosine hydroxylase (TH)-containing neurons and by reducing microglial reactivity and macrophage infiltration [6, 7]. In contrast, 6-hydroxydopamine-lesioned mice did not respond to CB_2_ receptor activation [8]. Such differences were recently confirmed by Concannon and coworkers [9] who compared the elevation of CB_2_ receptors in LPS-lesioned rats, which was paralleled by increased microglial activation, with the poor response found in rodents lesioned with 6-hydroxydopamine. However, work conducted by Ternianov and coworkers [10] supported a role of CB_2_ receptors in 6-hydroxydopamine-lesioned mice too, as they found that mice overexpressing CB_2_ receptors were more protected against 6-hydroxydopamine-induced nigrostriatal damage.

The anti-inflammatory potential of cannabinoids in PD has been recently reinforced with the possibility that some of them can also bind and activate specific receptor types of the peroxisome proliferator-activated receptor (PPAR) family such as PPARγ [11, 12]. Such nuclear receptors have long been involved in the control of neuroinflammatory responses [13], whereas specific non-cannabinoid PPARγ activators (e.g., thiazolidinediones) have been found to be active in experimental models of PD and have entered recently in clinical investigation [14]. Some endocannabinoids (e.g., anandamide and 2-arachidonoylglycerol) and their related signaling lipids (e.g., palmitoylethanolamide, oleylethanolamide), as well as different phytocannabinoids and their derivatives, have been found to exert PPARγ-mediated anti-inflammatory activity [12, 15, 16]. We have recently designed, synthesized, and characterized different phytocannabinoid derivatives, in particular a series of quinone derivatives of cannabigerol (CBG) that behave as PPARγ activators [17–19], while retaining the lack of CB_1_/CB_2_ activity of their phytocannabinoid template. For this study, we were particularly interested in one non-thiophilic CBG quinone derivative, so-called VCE-003.2, whose ability to activate PPARγ [19] enables this compound to serve as an anti-inflammatory and a neuroprotectant in LPS-lesioned mice, the experimental model of PD that better reproduces inflammation as a pathogenic event in this disease. We have investigated VCE-003.2 in this in vivo PD model following previous research conducted in murine models of Huntington’s disease [19], which confirmed its activity at the PPARγ and its capability to cross the blood-brain barrier after systemic administration. In addition, we have extended this research with some in vitro experiments useful to confirm the anti-inflammatory profile of VCE-003.2 (cultured BV2 cells stimulated with LPS) or its neuroprotective effects (cultured M-213 neuronal cells incubated with conditioned media generated from cultured BV2 cells stimulated with LPS). In all these experimental approaches, the possible contribution of PPARγ activation has been investigated using selective inhibitors of these nuclear receptors. However, the lack of activity of classic PPARγ antagonists (e.g., T0070907, GW9662) against VCE-003.2 effects in the in vitro studies and the recent identification of a functional alternative binding site for PPARγ ligands that does not overlap with the canonical binding site used by glitazones [20] prompted us to investigate whether VCE-003.2 binds to this receptor at this different site using docking and transcriptional analyses. In an additional experiment presented as supplementary data (see Additional file 3), we have also investigated whether the cannabinoid receptor-independent antioxidant profile of VCE-003.2 enables this compound to also serve as neuroprotectant in 6-hydroxydopamine-lesioned mice, which is characterized, as mentioned above, by mitochondrial dysfunction and oxidative stress but having a poor inflammatory response. In this model, other antioxidant phytocannabinoids (Δ^9^-tetrahydrocannabinol, cannabidiol, Δ^9^-tetrahydrocannabivarin) have been shown to preserve nigrostriatal dopaminergic neurons by antioxidant mechanisms independent of cannabinoid receptors [7, 8, 21]. With this study in whole, we expect to add experimental support to the idea that a pharmaceutical formulation, using a pleiotropic cannabinoid derivative targeting PPARγ receptors, and perhaps other relevant targets for PD, may be of great interest to reduce inflammation and oxidative stress, as well as to enhance neuronal integrity in PD.

## Methods

### Synthesis and receptor characterization of VCE-003.2

The quinone derivative of CBG (6-(3,7)-dimethyl-octa-2,6-dienyl)-5-hydroxy-3-pentyl-2-ethylamino-[1,4]benzoquinone), so-called VCE-003.2, was synthesized as described previously [19]. Its activity as a PPARγ activator was also previously characterized [19]. To confirm that, as expected, VCE-003.2 has no affinity at the CB_1_ and the CB_2_ receptors, we conducted radioligand binding assays using membranes purified from cells transfected with human CB_1_ or CB_2_ receptors (RBHCB1M400UA and RBXCB2M400UA; Perkin-Elmer Life and Analytical Sciences, Boston, MA, USA). The protein concentration was 8 μg/well for the CB_1_ receptor membranes and 4 μg/well for those of the CB_2_ receptor. The binding buffer was 50 mM Tris-Cl, 5 mM MgCl_2_, 2.5 mM EDTA, and 0.5 mg/mL bovine serum albumin (pH = 7.4) for CB_1_ and 50 mM TrisCl, 5 mM MgCl_2_, 2.5 mM EGTA, and 1 mg/mL bovine serum albumin (pH = 7.5) for CB_2_. The radioligand was [^3^H]-CP55940 (Perkin-Elmer) used at a concentration of membrane K_D_ × 0.8 nM, and the final incubation volume was 200 μl for CB_1_ and 600 μl for CB_2_. Ninety-six-well plates and the tubes necessary for the experiment were previously siliconized with Sigmacote (Sigma-Aldrich, Madrid, Spain). Membranes were resuspended in the corresponding buffer and were incubated (90 min at 30 °C) with the radioligand and VCE-003.2 at a high concentration (40 μM) with the purpose to determine the percentage of radioligand displacement. Non-specific binding was determined with 10 μM WIN55212-2 and total radioligand binding by incubation with the membranes in absence of VCE-003.2. Filtration was performed by a Harvester® filtermate (Perkin-Elmer) with Filtermat A GF/C filters pretreated with polyethylenimine 0.05%. After filtering, the filter was washed nine times with a binding buffer and dried and a melt-on scintillation sheet (Meltilex™ A, Perkin-Elmer) was melted onto it. Then, radioactivity was quantified by a liquid scintillation spectrophotometer (Wallac MicroBeta Trilux, Perkin-Elmer). In the case of both CB_1_- and CB_2_-transfected membranes, radioligand displacement at these conditions was always lower than 50%, then indicating negligible activity at both cannabinoid receptor types with Ki values in the micromolar range (> 40 μM; data from at least 3 experiments performed in triplicate for each point).

### Cultures of BV2 cells and M-213 neuronal cells

In a first experiment, mouse BV2 microglial cells (kindly provided by Dr. Carmen Guaza, Instituto Cajal, CSIC) were cultured in Dulbecco’s modified Eagle’s medium (DMEM, Lonza, Verviers, Belgium) supplemented with 10% fetal bovine serum (FBS, Sigma-Aldrich, Madrid, Spain), 2 mM UltraGlutamine, and antibiotics (Lonza, Verviers, Belgium) in a humidified atmosphere of 5% CO_2_ at 37 °C. Cells were plated at a density of 45 × 10^4^ cells per well in 12-well culture plates and incubated in DMEM with a reduction of FBS to 1%. Three hours later, cells were treated with 0.5 μg/ml LPS (from *Escherichia coli* 055:B5, Sigma-Aldrich, Madrid, Spain), alone or in combination with VCE-003.2, used at a concentration of 5 μM (selected from previous concentration–response studies), and added 1 h before LPS. Twenty hours after the addition of LPS, media were removed and used for the analysis of tumor necrosis factor-α (TNF-α) and interleukin-1β (IL-1β) levels using commercial ELISA kits (ref. MTA00B, R&D Systems, Minneapolis, MN, USA for TNF-α, and ref. MLB00C, R&D Systems, Minneapolis, MN, USA for IL-1β), whereas cell pellets were collected for analyzing protein levels by the Lowry method, which were used to normalize the cytokine data. In a follow-up experiment, the procedure for cell plating, incubation, and treatment with LPS and VCE-003.2 described above was repeated again, but an additional experimental group consisting of cells treated with LPS (0.5 μg/ml), VCE-003.2 (5 μM), and GW9662 (10 μM; Abcam, Cambridge, UK) was added. Twenty hours after the addition of LPS, cell pellets were collected for the qPCR analysis of TNF-α, IL-1β, inducible nitric oxide synthase (iNOS), and cyclooxygenase-2 (COX-2).

In a second experiment, cultured BV2 cells were maintained in DMEM (Lonza, Verviers, Belgium) supplemented with 10% fetal bovine serum (FBS, Sigma-Aldrich, Madrid, Spain), 2 mM UltraGlutamine, and antibiotics (Lonza, Verviers, Belgium) in a humidified atmosphere of 5% CO_2_ at 37 °C. For experiments, cells were plated at a density of 2 × 10^5^ cells per well in 6-well culture plates and incubated in DMEM with a reduction of FBS to 1%. Three hours later, cells were treated with 0.5 μg/ml LPS (from *Escherichia coli* 055:B5, Sigma-Aldrich, Madrid, Spain). Twenty-four hours after the addition of LPS, media were removed to be added to cultures of the rat M-213 striatal cell line (kindly provided by Dr. WJ Freed, National Institute on Drug Abuse, Bethesda, MD, USA) to induce cell death following a procedure described previously [22]. To this end, M-213 cells were maintained in DMEM supplemented with 10% FBS, 2 mM UltraGlutamine, and 1% antibiotics (Lonza, Verviers, Belgium) under a humidified 5% CO_2_ atmosphere at 37 °C. For cytotoxicity experiments, cells were seeded at 50,000 cells/well in 24-well plates and maintained under a humidified atmosphere (5% CO_2_) at 37 °C overnight. Afterwards, normal medium was completely replaced by the conditioned media generated in BV2 cell experiments, and then, M-213 cells were treated with the vehicle (0.1% DMSO) or with three different concentrations of VCE-003.2 (0.1, 0.5, and 1 μM; selected according to our previously published study [19], which described an IC50 = 1.2 μM for the binding of VCE003.2 to PPARγ receptors), added alone or combined with the selective PPARγ inhibitor T0070907 (10 μM; Cayman Chemical, Ann Arbor, Michigan, USA). Rosiglitazone (RGZ) (20 μM, according to [23] and laboratory experience; Sigma-Aldrich, Madrid, Spain) was also added, alone or combined with T0070907 (10 μM), as a positive control for PPARγ activation. Cells were incubated for 40 h before the neuronal death was analyzed with the MTT assay (Panreac AppliChem., Barcelona, Spain). The data were normalized in relation with a control group consisting in M-213 cells exposed to conditioned media generated by BV2 cells in absence of LPS.

### Mesenchymal stem cell differentiation

Human mesenchymal stem cells (MSCs) derived from bone marrow were obtained as previously described [19]. Cells were seeded in α-MEM containing 15% FCS, 2 mM UltraGlutamine, 1 ng/ml bFGF, and antibiotics, and adipogenic differentiation was performed as described [19]. Treatment with RGZ (1 μM) and VCE-003.2 (1 μM) in the presence and the absence of T0070907 (5 μM) started at the same time as the differentiation process. After a week of differentiation, the mRNA expression for PPARγ isoform 2 (PPARγ2), lipoprotein-lipase (LPL), CCAAT/enhancer-binding protein-α (CEBPA), adiponectin (ADIPOQ), and fatty acid-binding protein 4 (FABP4) was analyzed as described [19].

### PPARγ transcriptional assays

To analyze PPARγ transcriptional activity, HEK-293T cells were cultured in 24-well plates and transiently co-transfected with the expression vector GAL4-PPARγ and the luciferase reporter vectors GAL4-luc (firefly luciferase) and pRL-CMV (renilla luciferase) using Roti©-Fect (Carl Roth, Karlsruhe, Germany). After stimulation, the luciferase activities were quantified using Dual-Luciferase Assay (Promega, Madison, WI, USA).

### Immunoblotting

Whole cell extracts were obtained by lysing the cells in NP-40 buffer (50 mM Tris-HCl pH 7.5, 150 mM NaCl, 10% glycerol, and 1% NP-40) supplemented with protease and phosphatase inhibitors. Lysate concentrations were determined by the Bradford assay (Bio-Rad Laboratories, Hercules, CA, USA). Proteins (30 μg/lane) were separated by SDS-PAGE, transferred onto PVDF membranes, and blocked with PBS-T (PBS + 0.1% Tween-20) containing 5% non-fat dry milk for 30 min at room temperature. Incubation with anti-PPARγ (ref. C26H12, Cell Signaling Technology, Beverly, MA, USA) and anti-α-actin (ref. AC-74, Sigma-Aldrich, Madrid, Spain) was performed overnight at 4 °C, and washed membranes were incubated with appropriate secondary antibodies coupled to horseradish peroxidase that were detected by an enhanced chemiluminescent reagent.

### Animals and surgical lesions

Male C57BL/6 mice were housed in a room with a controlled photoperiod (06:00–18:00 light) and temperature (22 ± 1 °C). They had free access to standard food and water and were used at adult age (3- to 4-month-old; 25–30 g weight). All experiments were conducted according to European guidelines (directive 2010/63/EU) and approved by the “Comité de Experimentación Animal” of our university (ref. CEA-UCM 56/2012). For in vivo experiments, mice were anesthesized (ketamine 40 mg/kg + xylazine 4 mg/kg, i.p.) and subjected to unilateral injections of *S. Minnesota* LPS (Sigma-Aldrich, Madrid, Spain) into two points of the right striatum following the procedure developed by Hunter et al. [24]. We used the following stereotaxic coordinates from bregma: + 1.18 mm AP, − 1.5 mm ML, and − 3.5 mm DV, as well as − 0.34 mm AP, − 2.5 mm ML, and − 3.2 mm DV (see details in [24]). At each intrastriatal coordinate, 5 μg of LPS in a volume of 1 μl of saline was injected slowly (0.5 μl/30 s) and the needle was left in place for 5 min before being slowly withdrawn. This avoids generating reflux and a rapid increase in intracranial pressure. Controls were sham-operated and injected with 1 μl of saline using the same coordinates. After the application of LPS or saline, mice were also subjected to pharmacological treatments as described in the following section. The lesions were generated using unilateral administration, the advantage of which is that contralateral structures serve as controls for the different analyses.

### Pharmacological treatments and sampling

LPS-lesioned mice were distributed in three groups and administered i.p. with 10 mg/kg of VCE-003.2, alone or in combination with 5 mg/kg of the PPARγ antagonist T0070907 (Cayman Chemical, Ann Arbor, Michigan, USA) [25] or vehicle [DMSO (3.3%) + Tween 20 (2%) + saline (94.7%)]. The experiment included a fourth group consisting of sham-operated mice also treated with DMSO-Tween 20-saline. The treatment was initiated approximately 16 h after the LPS lesion and was repeated daily for 21 days. One day after the last injection, mice were killed by rapid and careful decapitation and their brains were rapidly removed and frozen in 2-methylbutane cooled in dry ice and stored at − 80 °C for subsequent immunohistochemical analysis in the substantia nigra and qPCR analysis in the striatum.

### Real-time qRT-PCR analysis

Brain coronal slices (around 500 μm thick) were made at levels containing the striatum, according to Palkovits and Brownstein Atlas [26]. Subsequently, such structure was dissected and used for qRT-PCR analysis. Cell pellets from the in vitro experiments were also used for qRT-PCR analysis. Total RNA was isolated from the different samples using SurePrep RNA/Protein Purification Kit (Fisher Bioreagents, Madrid, Spain). The total amount of RNA extracted was quantitated by spectrometry at 260 nm and its purity from the ratio between the absorbance values at 260 and 280 nm. After genomic DNA was removed (to eliminate DNA contamination), single-stranded complementary DNA was synthesized from up to 1 μg of total RNA using the commercial kits Rneasy Mini Quantitect Reverse Transcription (Qiazen, Hilgen, Germany) and iScript™ cDNA Synthesis Kit (Bio-Rad, Hercules, CA, USA). The reaction mixture was kept frozen at − 20 °C until enzymatic amplification. Quantitative RT-PCR assays were performed using TaqMan Gene Expression Assays (Applied Biosystems, Foster City, CA, USA) to quantify mRNA levels for TNF-α (ref. Mm99999068_m1), IL-1β (ref. Mm00434228_m1), iNOS (ref. Mm01309902_m1), and COX-2 (ref. Mm00478372_m1), using GAPDH expression (ref. Mm99999915_g1) as an endogenous control gene for normalization. The PCR assay was performed using the 7300 Fast Real-Time PCR System (Applied Biosystems, Foster City, CA, USA), and the threshold cycle (Ct) was calculated by the instrument’s software (7300 Fast System, Applied Biosystems, Foster City, CA, USA). Expression levels were calculated using the 2^−ΔΔCt^ method.

### Immunohistochemical procedures

Brains were sliced in coronal sections (containing the substantia nigra) in a cryostat (30 μm thick) and collected on antifreeze solution (glycerol/ethylene glycol/PBS; 2:3:5) and stored at − 20 °C until used. Sections were mounted on gelatin-coated slides and, once adhered, washed in 0.1 M potassium PBS (KPBS) at pH 7.4. Then, endogenous peroxidase was blocked by 30-min incubation at room temperature in a peroxidaseblocking solution (Dako Cytomation, Glostrup, Denmark). After several washes with KPBS, sections were incubated overnight at room temperature with the following primary antibodies: (i) rabbit polyclonal anti-TH (Chemicon-Millipore, Temecula, CA, USA) used at 1/400; (ii) rabbit polyclonal anti-Iba-1 antibody (Wako Chemicals, Richmond, VA, USA) used at 1/300; or (iii) monoclonal rat anti-mouse Cd68 antibody (AbD Serotec, Oxford, UK) used at 1/200. Dilutions were carried out in KPBS containing 5% normal horse serum and 0.1% Triton X-100 (Sigma Chem., Madrid, Spain). After incubation, sections were washed in KPBS, followed by incubation with the corresponding biotinylated secondary antibody (1/400) (Vector Laboratories, Burlingame, CA, USA) for 1 h at room temperature. Avidin–biotin complex (Vector Laboratories, Burlingame, CA, USA) and 3,3′-diaminobenzidine substrate–chromogen system (Dako Cytomation, Glostrup, Denmark) were used to obtain a visible reaction product. Negative control sections were obtained using the same protocol with omission of the primary antibody. A Leica DMRB microscope and a DFC300FX camera (Leica, Wetzlar, Germany) were used for the observation and photography of the slides, respectively. For quantification of the intensity of TH, Iba-1, or CD68 immunostaining either in the substantia nigra (both ipsilateral and contralateral sides), we used the NIH Image Processing and Analysis software (ImageJ; NIH, Bethesda, MD, USA) using 4–5 sections, separated approximately by 200 μm, and observed with × 5–20 objectives depending on the method and the brain area under quantification. In all sections, the same area of the substantia nigra *pars compacta* was analyzed. Analyses were always conducted by experimenters who were blinded to all animal characteristics. Data were expressed as percentage of immunostaining intensity in the ipsilateral (lesioned) side over the contralateral (non-lesioned) side.

Immunofluorescence was used for a double-labeling analysis with TH and Cd68 in sections containing the substantia nigra pars compacta. After pre-incubation for 20 min with Tris-buffered saline with 1% Triton X-100 (pH 7.5), sections were sequentially incubated overnight at 4 °C with a monoclonal rat anti-mouse Cd68 antibody (AbD Serotec, Oxford, UK) used at 1/200, followed by washing in a Tris-buffered saline and a new incubation (at 37 °C for 2 h) with an anti-rat secondary antibody made in donkey (1/200) conjugated with Alexa 488 (Life Technologies-Thermofisher Scientific, Waltham, MA, USA) rendering green fluorescence. Sections were then washed again and incubated overnight at 4 °C with a polyclonal rabbit anti-TH (Chemicon-Merck Millipore, Darmstadt, Germany) used at 1/200, again followed by washing in a Tris-buffered saline and a new incubation (at 37 °C for 2 h) with an anti-rabbit secondary antibody made in goat (1/200) conjugated with Alexa 546 (Invitrogen, Carlsbad, CA, USA) rendering red fluorescence. Sections were counter-stained with nuclear stain TOPRO-3-iodide (Molecular Probes, Eugene, OR, USA) to visualize cell nuclei. A SP5 Leica confocal microscope was used for slide observation and photography.

### Docking analysis

Ligand docking was performed using the *AutoDock4* [27] and the *Vina* software [28] with the virtual screening tools *PyRx* [29] and PyMOL [30]. The receptor model used was the PDB reference (RCSB Protein Data Bank accession code) 2Q59 and 3B0R [20], and 4EMA [31]. The search space for the docking, around the receptor molecule surface, was set according to previous findings about several binding sites for different ligands. Once analysis has been performed, *AutoDock Vina* provides the estimated binding affinity value, which is the sum of the intermolecular energy, due to the interaction between both molecules, and the torsional free-energy penalty, due to the conformation adopted by these molecules to properly fit the interaction surface. A negative value indicates that the bond is thermodynamically stable, whereas a positive value means instability. Search space for the docking was set around the binding sites described previously [20].

### Data analysis

Data were subjected to the one-way analysis of variance followed by the Student–Newman–Keuls, Tukey, or the Bonferroni multiple comparison tests.

## Results

### Determination of CB_1_/CB_2_ receptor binding for VCE-003.2

VCE-003.2 was synthesized as described in a previous study [19], in which its pharmacodynamic properties, in particular its activity as a PPARγ activator, were already investigated using transcriptional assays [19]. VCE-003.2 is a derivative of CBG, a non-psychoactive cannabinoid present in the Cannabis plant that does not exhibit any activity at the CB_1_ receptor [32]. We have recently confirmed that CBG binds the CB_2_ receptor also with a poor affinity in the micromolar range [17]. Therefore, it was expected that its quinone derivative VCE-003.2 has no affinity at both CB_1_ and CB_2_ receptors. To confirm that, we conducted radioligand binding assays using membranes purified from cells transfected with human CB_1_ or CB_2_ receptors. Our data demonstrated that a high concentration of VCE-003.2 (40 μM) displaced radioligand binding to both CB_1_ and CB_2_ receptors with a percentage in both cases lower than 50%, then indicating negligible affinity for both receptors with predicted Ki always > 40 μM in both cases.

### Anti-inflammatory and neuroprotective effects of VCE-003.2 in LPS-lesioned mice

**Fig. 1:**
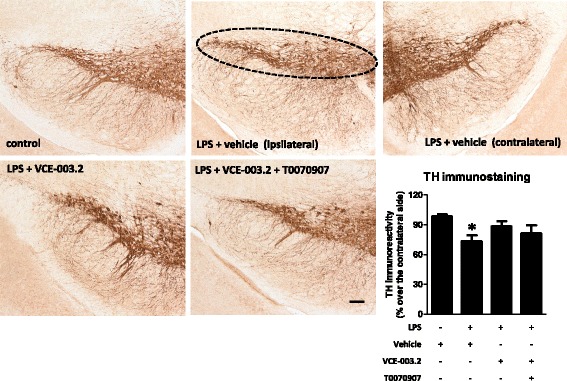
Intensity of the immunostaining for TH (bottom right panel) measured in a selected area of the substantia nigra pars compacta of adult male mice at 3 weeks of being subjected to an intrastriatal injection of LPS and daily treated with VCE-003.2 (10 mg/kg), alone or combined with T0070907 (5 mg/kg), or vehicle. Immunoreactivity values are measures in the lesioned side over the non-lesioned side and correspond to means ± SEM of 4–6 subjects *per* group. Data were assessed by the one-way analysis of variance followed by the Student–Newman–Keuls test (*p < 0.05 versus vehicle-treated control mice). Representative immunostaining images for each experimental group, with indication of the approximate area quantified, are shown in the top and left panels (scale bar = 100 μm)

**Fig. 2:**
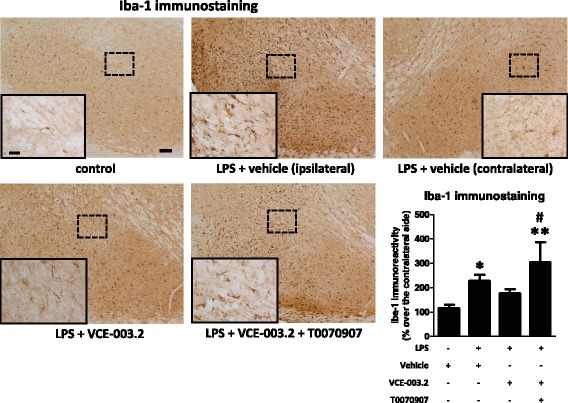
Intensity of the immunostaining for Iba-1 (bottom right panel) measured in a selected area of the substantia nigra pars compacta of adult male mice at 3 weeks of being subjected to an intrastriatal injection of LPS and daily treated with VCE-003.2 (10 mg/kg), alone or combined with T0070907 (5 mg/kg), or vehicle. Immunoreactivity values are measures in the lesioned side over the non-lesioned side and correspond to means ± SEM of 4–6 subjects *per* group. Data were assessed by the one-way analysis of variance followed by the Student–Newman–Keuls test (*p < 0.05, **p < 0.01 versus vehicle-treated control mice; ^#^*p* < 0.05 versus VCE-003.2-treated LPS-lesioned mice). Representative Iba-1 immunostaining images for each experimental group are shown in the top and left panels (scale bar = 100 μm), including a specific inlet showing the morphological characteristics of Iba-1-labeled cells (scale bar = 25 μm)

**Fig. 3:**
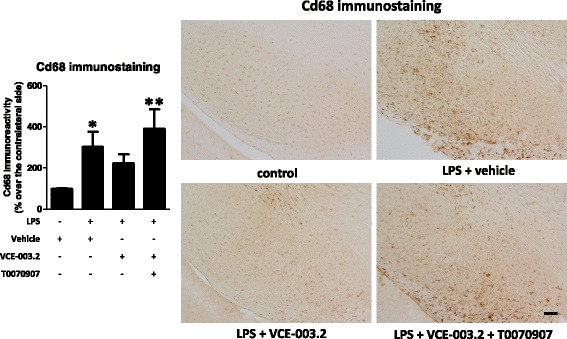
Intensity of the immunostaining for Cd68 (left panel) measured in a selected area of the substantia nigra pars compacta of adult male mice at 3 weeks of being subjected to an intrastriatal injection of LPS and daily treated with VCE-003.2 (10 mg/kg), alone or combined with T0070907 (5 mg/kg), or vehicle. Immunoreactivity values are measures in the lesioned side over the non-lesioned side and correspond to means ± SEM of 4–6 subjects *per* group. Data were assessed by the one-way analysis of variance followed by the Student–Newman–Keuls test (*p < 0.05, **p < 0.01 versus vehicle-treated control mice). Representative Cd68 immunostaining images for each experimental group are shown in the right panels (scale bar = 100 μm)

Given the activity of VCE-003.2 at the PPARγ receptors, which is an important factor involved in the control of inflammation, we wanted to evaluate its anti-inflammatory/neuroprotective properties in a typically in vivo inflammatory model of PD, LPS-lesioned mice. It is likely that, in the absence of CB_2_ receptor activity for VCE-003.2, such beneficial effects, if they exist, should be possibly mediated by enhancing PPARγ signaling. First, we analyzed TH immunostaining in the substantia nigra pars compacta and demonstrated a reduction of immunoreactivity in LPS-lesioned mice (*F*(3,17) = 5.21, *p* < 0.05; see Fig. 1) in concordance with the previous data [6, 7], and evident here comparing the lesioned ipsilateral side with the non-lesioned contralateral side (Fig. 1). In addition to these quantitative data, the morphological analysis of the immunostained substantia nigra showed an evident reduction in neuropil (Fig. 1), possibly reflecting neurite losses in nigral neurons. The reduction in immunoreactivity levels and also the deteriorated aspect of neuropil in nigral neurons were reversed by the treatment with VCE-003.2 (Fig. 1). In addition, we also found a lowering effect of VCE-003.2 on the intense microgliosis provoked by LPS and revealed by Iba-1 (*F*(3,18) = 6.76, *p* < 0.005; see Fig. 2) and Cd68 (*F*(3,19) = 5.03, *p* < 0.05; see Fig. 3) immunostaining in the substantia nigra. Such microgliosis was evident not only by the elevated immunoreactivity but also by the presence of microglial cells (labeled with Iba-1) with ameboid aspect (activated state) compared with the ramified form (quiescent state) currently found in controls (Fig. 2). The treatment with VCE-003.2 changed this activated morphology towards a classic resting aspect (Fig. 2). In addition, using a double immunofluorescence analysis for TH and Cd68, we confirmed that this reactive microgliosis was specifically located inside and surrounding the substantia nigra pars compacta and associated with the loss of TH-positive neurons. This analysis is presented as a supplementary figure (see Additional file 1) and demonstrates the proximity of TH and Cd68 labeling, but the expected lack of any overlapping.

**Fig. 4:**
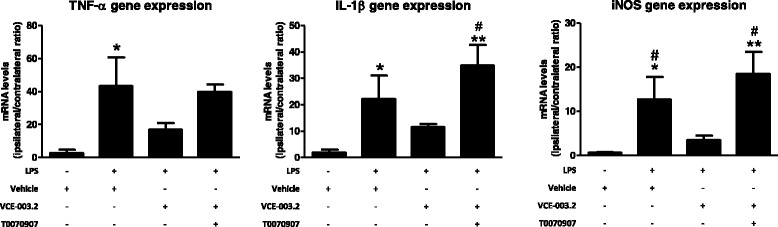
mRNA levels for TNF-α, IL-1β, and iNOS in the striatum of adult male mice at 3 weeks of being subjected to an intrastriatal injection of LPS and daily treated with VCE-003.2 (10 mg/kg), alone or combined with T0070907 (5 mg/kg), or vehicle. Values are the ratio between the lesioned side and the non-lesioned side and correspond to means ± SEM of 4–6 subjects *per* group. Data were assessed by the one-way analysis of variance followed by the Student–Newman–Keuls test (*p < 0.05, **p < 0.01 versus vehicle-treated control mice; ^#^*p* < 0.05 versus VCE-003.2-treated LPS-lesioned mice)

We also analyzed by qPCR several proinflammatory markers typical of the activated microglial cells recruited at the lesioned ipsilateral striatum, always compared to the non-lesioned contralateral side. As expected, we detected a strong elevation in the expression of two proinflammatory cytokines TNF-α (*F*(3,19) = 4.375, *p* < 0.05; Fig. 4) and IL-1β (*F*(3,19) = 6.472, *p* < 0.005; Fig. 4), as well as in the proinflammatory enzyme iNOS (*F*(3,19) = 6.808, *p* < 0.005; Fig. 4) in the striatum of LPS-lesioned mice, all of them compatible with the microglial activation found in these mice (Figs. 2 and 3). The elevation in iNOS was attenuated by the treatment with VCE-003.2, with differences between vehicle- and VCE-003.2-treated LPS-lesioned mice being statistically significant (Fig. 4). Moreover, the treatment of LPS-lesioned mice with VCE-003.2, although did not reach a statistically significant difference compared to vehicle-treated LPS-lesioned animals in TNF-α and IL-1β, provoked that the values for both cytokines are no longer statistically different compared to control mice, thus supporting a modest but evident effect (Fig. 4).

The most interesting was that these effects and also those observed with VCE-003.2 on the microglial activation (Iba-1 and Cd68 immunostaining) disappeared when the CBG derivative was co-administered with the PPARγ antagonist T0070907 (Figs. 2, 3, and 4), then supporting the involvement of this nuclear receptor in VCE-003.2 effects. The only effect of VCE-003.2 that was not reversed by T0070907 in a statistically significant manner was the recovery in TH immunostaining, although the pattern of changes showed the expected tendency (Fig. 1). It is also important to remark that, in some cases, e.g., Iba-1 and Cd68 immunostaining and IL-1β and iNOS gene expression, the values reached after the blockade of PPARγ receptors in VCE-003.2-treated LPS-lesioned mice were even elevated when compared to the vehicle-treated LPS-lesioned mice (Figs. 2, 3, and 4). This may suggest the existence of an endogenous basal tone activating PPARγ, whose apparent beneficial effects would be eliminated only when these receptors are blocked. Such idea would be concordant with previous data showing that GW9662, another irreversible PPARγ inhibitor, was toxic for dopaminergic neurons in MPTP-treated mice [33].

This evaluation of the neuroprotective effects of VCE-003.2 in in vivo models of PD also included experiments in an additional model of parkinsonism, mice lesioned with 6-hydroxydopamine, which, contrarily to LPS-lesioned mice, is a model with poor inflammation and glial reactivity and mainly based on oxidative stress derived from mitochondrial dysfunction. These data are presented as a supplementary material (see Additional file 3).

### Anti-inflammatory effects of VCE-003.2 against a LPS insult in cultured BV2 cells

**Fig. 5:**
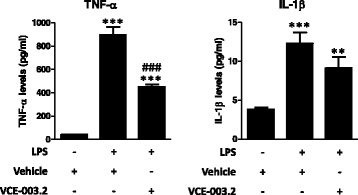
Levels of TNF-α and IL-1β measured in the media of cultured BV2 cells exposed to LPS (0.5 μg/ml) in the presence or absence of VCE-003.2 (5 μM). Values are means ± SEM of at least 4 independent experiments each performed in triplicate. Data were assessed by the one-way analysis of variance followed by the Student–Newman–Keuls test (**p < 0.01, ***p < 0.005 versus control cells (no treated with LPS); ^###^*p* < 0.005 versus cells treated with LPS)

**Fig. 6:**
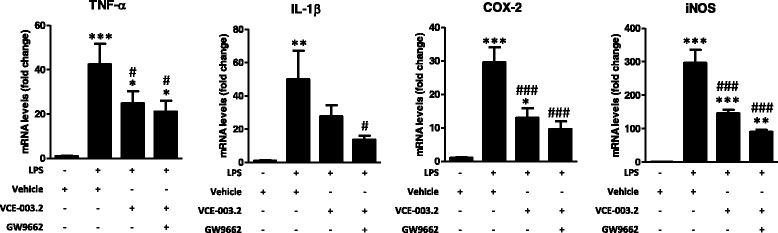
mRNA levels for TNF-α, IL-1β, COX-2, and iNOS measured in the cell pellets of cultured BV2 cells exposed to LPS (0.5 μg/ml) in the presence or absence of VCE-003.2 (5 μM) and/or GW9662 (10 μM). Values are means ± SEM of at least 7 independent experiments each performed in duplicate. Data were assessed by the one-way analysis of variance followed by the Student–Newman–Keuls test (*p < 0.05, **p < 0.01, ***p < 0.005 versus control cells (no treated with LPS); ^#^*p* < 0.05, ^###^*p* < 0.005 versus cells treated with LPS)

Next, we used some in vitro approaches to further investigate the benefits found with VCE-003.2 in the inflammatory in vivo model of PD. We investigated first the anti-inflammatory profile of VCE-003.2 in cultured BV2 cells exposed to LPS. On the one hand, VCE-003.2 was able to attenuate the LPS-induced release of TNF-α (*F*(2,43) = 104.4, *p* < 0.0001; Fig. 5) and reduced the *p* values compared to controls in the case of IL-1β (*F*(2,44) = 18.72, *p* < 0.0001; Fig. 5), with both cytokines being measured in culture media using ELISA kits. On the other hand, this was associated with similar changes measured by qPCR in cell pellets, with elevations after LPS which were attenuated after the treatment with VCE-003.2 for TNF-α (*F*(3,27) = 8.327, *p* < 0.001; Fig. 6) and for two proinflammatory enzymes like COX-2 (*F*(3,27) = 16.10, *p* < 0.0001; Fig. 6) and iNOS (*F*(3,27) = 35.95, *p* < 0.0001; Fig. 6). Again, in the case of IL-1β (*F*(3,27) = 5.05, *p* < 0.01; Fig. 6), the effect of VCE-003.2 consisted in a reduction in *p* values compared to controls (Fig. 6). However, these effects of VCE-003.2 effects were not inhibited by the PPARγ antagonist GW9662; in fact, they were apparently enhanced in the presence of this antagonist (Fig. 6). We do not believe that this result may be due to the change in the PPARγ antagonist because, as mentioned above, GW9662 is other synthetic irreversible PPARγ antagonist, which, as T0070907, covalently binds to Cys285 into the PPARγ ligand-binding pocket (LBP). The only difference between both antagonists is a single nitrogen substitution, so that both compounds can be indistinctly used as a tool to investigate PPARγ activation by different ligand activators. Hence, the fact that the effects of VCE-003.2 were not reversed by GW9662, which would presumably occur with T0070907 too, might indicate that, in LPS-treated BV2 cells, VCE-003.2 might act also through PPARγ-independent mechanisms, and this may be the consequence of the well-known LPS-induced downregulation of these receptors found in BV2 cells [34] and also in macrophages [35]. However, we do not believe that this may happen in our study, as we have evidence that both RGZ and VCE-003.2 increase the expression of PPARγ, which, in general, is known to be upregulated by PPARγ ligand agonists, counteracting the repressive effect of LPS on the expression of this nuclear receptor (see Additional file 2). As regards the enhancing action of GW9662 on VCE-003.2 effects in LPS-treated BV2 cells, we believe that they may be related to an additive interaction of both at the PPARγ LBP derived from a potential activity of GW9662 as partial agonist too. This has been previously described for different biological activities of this class of antagonists [36, 37], whereas, in our hands, both GW9662 and T0070907 exert PPARγ transcriptional activity in a Gal4-Luc system, although to a much lesser extent than RGZ and VCE-003.2 (data not shown).

### Neuroprotective effects of VCE-003.2 in M-213 neuronal cells exposed to conditioned media generated by cultured BV2 cells exposed to LPS

**Fig. 7:**
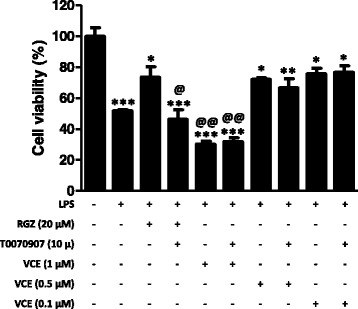
Cell viability measured in cultured M-213 cells treated with different concentrations of VCE-003.2 (0.1, 0.5, and 1.0 μM) or RGZ (20 μM) in the presence or the absence of T0070907 (10 μM) and exposed to conditioned media generated from LPS-stimulated BV2 cells. The control (100% of cell viability) consisted in M-213 cells that were exposed to conditioned media in the absence of LPS. Values are means ± SEM of at least 4 independent experiments each performed in duplicate. Data were assessed by the one-way analysis of variance followed by the Student–Newman–Keuls test (*p < 0.05, **p < 0.01, ***p < 0.005 compared to the control cells (incubated with conditioned media no generated with LPS); ^@^*p* < 0.05, ^@@^*p* < 0.01 versus cells treated with RGZ, as well as versus cells treated with VCE-003.2 (0.1 and 0.5 μM) combined or not with T0070907 (incubated with conditioned media generated with LPS))

The second in vitro approach we used to further explore the neuroprotective properties of VCE-003.2 in PD consisted in investigating its capability to protect cultured M-213 neuronal cells against death induced by exposure to conditioned media generated from cultured BV2 cells overexposed to LPS. This is a classic method to investigate neuroprotection against inflammation-driven neurotoxicity [22]. First, we wanted to evaluate different concentrations of VCE-003.2 and what we found was the classic dual effect of cannabinoids, with low concentrations (0.1–0.5 μM) preserving M-213 cells against the inflammatory insult, to a similar extent than RGZ used as a positive control for PPARγ, whereas higher concentrations, but not particularly high (1 μM), resulted toxic (*F*(9,38) = 24.95, *p* < 0.0001; Fig. 7). This was mostly similar to the results found with VCE-003.2 in cultured neural progenitor cells, in which toxicity was evident from a concentration of 2 μM [19]. The three concentrations of VCE-003.2 and RGZ were examined also in the presence of the PPARγ receptor inhibitor T0070907 in order to determine whether their effects were mediated by activation of these receptors. However, whereas the effect of RGZ was completely reversed by the PPARγ receptor inhibitor T0070907, this inhibitor was inactive against the effect of VCE-003.2 at the two protective concentrations (Fig. 7), then suggesting the contribution of mechanisms other than the canonic activation of PPARγ in these effects. The same happened with the toxic concentration (Fig. 7).

### VCE-003.2 binds to the canonical and alternative sites in the PPARγ LBP

**Fig. 8:**
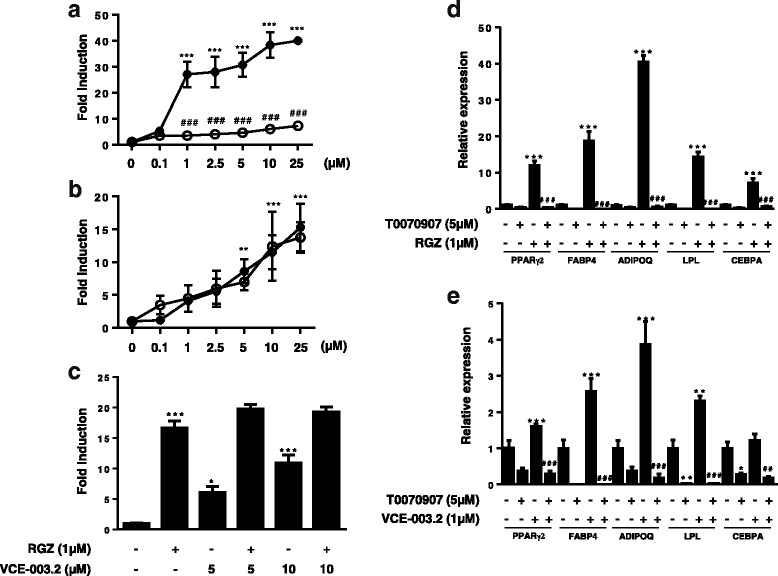
Effects of T0070907 on RGZ- and VCE-003.2-induced PPARγ transcriptional activity and MSCs differentiation. HEK-293T cells were transiently transfected with PPARγ-GAL4 plus GAL4-luc, pre-incubated with T0070907 (5 μM) for 15 min and then treated with increasing concentrations of either RGZ (**a**) or VCE-003.2 (**b**) for 6 h and luciferase activity measured in the cell lysates (open circles, PPARγ ligand; dark circles, PPARγ ligand plus T0070907). **c** Cells were transfected with the same pair of plasmids and treated with RGZ, VCE-003.2, or a combination of both compounds for 6 h and luciferase activity measured in the cell lysates. T0070907 prevented **d** RGZ- and **e** VCE-003.2-induced adipogenic differentiation in MSCs. The cells were differentiated in AM in the presence of RGZ and VCE-003.2 in the absence and the presence of T0070907, and adipogenic markers were characterized. Gene expression of adipogenic markers such as *PPARγ2*, *LPL*, *FABP4*, *CEBPA*, and *ADIPOQ* were measured after 7 days of differentiation. Data were assessed by the one-way analysis of variance followed by the Tukey test (*p < 0.05, **p < 0.01, ***p < 0.001 RGZ or VCE-003.2 compared to the control cells; ^**##**^*p* < 0.01, ^**###**^*p* < 0.001 RGZ or VCE-003.2 + T0070907 compared to RGZ- or VCE-003.2-treated cells)

We have previously shown that VCE-003.2 is a PPARγ partial agonist compared to RGZ [19]. PPARγ receptor ligands can induce a graded response such as full and partial agonism and antagonism that may depend on the mode of interaction and binding to the LBP [20, 38]. Thus, we were interested to study the potential functionality of the canonical and alternative PPARγ sites involved in the response to VCE-003.2. Luciferase reporter assays were used to measure PPARγ-dependent transcription, and RGZ (*F*(13,28) = 84.77, *p* < 0.0001; Fig. 8a) and VCE-003.2 (*F*(13,35) = 15.41, *p* < 0.005; Fig. 8b) showed increase in PPARγ transactivation. To study the participation of the alternative binding site, the cells were pre-incubated with T0070907 before treating the cells with the agonist ligands. T0070907 did not block the action of VCE-003.2 (Fig. 8b), but it was very effective to block RGZ-induced PPARγ transactivation (Fig. 8a) that is consistent with the fact that RGZ activates PPARγ by acting mainly through the canonical binding site [20]. To further analyze the effects of VCE-003.2 at this nuclear receptor, we studied the behavior of this compound in the presence of RGZ. To achieve this, GAL4-PPARγ/GAL4-luc co-transfected cells were pre-incubated with increasing concentrations of VCE-003.2, then treated with 1 μM RGZ. VCE-003.2 did not affect RGZ-induced PPARγ transactivation, thus suggesting that VCE-003.2 does not compete with RGZ for the same binding site at the LBP PPARγ (Fig. 8c).

Next, we also studied the action of VCE-003.2 in MSCs, which is a valid cell model to assess endogenous PPARγ function related to adipogenic differentiation. MSCs were differentiated to adipocytes in the presence of VCE-003.2 or RGZ with or without T0070907 for 7 days, as reflected the data of mRNA expression for the PPARγ-dependent genes PPARγ2, LPL, CEBPA, ADIPOQ, and FABP4 analyzed by RT-PCR. In the absence of an antagonist, both RGZ [PPARγ2: *F*(3,8) = 67.85, *p* < 0.0001; LPL: *F*(3,8) = 114.4, *p* < 0.0001; CEBPA: *F*(3,8) = 27.37, *p* = 0.0005; ADIPOQ: *F*(3,8) = 491.1, *p* < 0.0001; FABP4: *F*(3,8) = 50.45, *p* < 0.0001; Fig. 8d] and VCE-003.2 [PPARγ2: *F*(3,8) = 67.85, *p* < 0.0001; LPL: *F*(3,8) = 63.38, *p* < 0.001; CEBPA: *F*(3,8) = 16.29, *p* < 0,005; ADIPOQ: *F*(3,8) = 24.67, *p* = 0.0016; FABP4: *F*(3,8) = 50.45, *p* < 0.0001; Fig. 8e] upregulated the expression of these adipogenic-related genes, although to a different extent, thus confirming the differences between a full PPARγ agonist (RGZ) and a PPARγ modulator (VCE-003.2) [19]. T0070907 abrogate the effects of both RGZ (Fig. 8d) and VCE-003.2 (Fig. 8e) on the expression of PPARγ-dependent genes, indicating that VCE-003.2 is also functional through the canonical LBP.

**Fig. 9:**
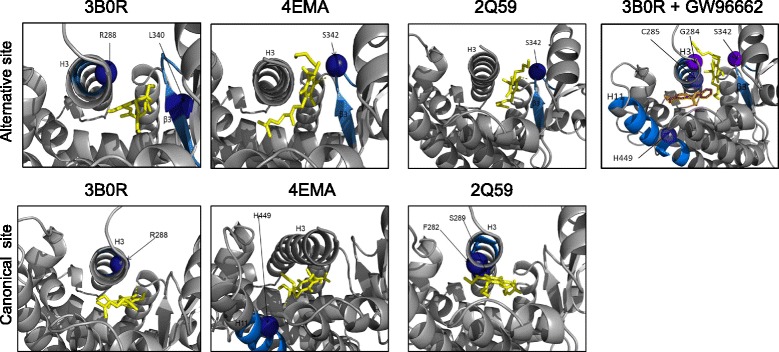
PPARγ LBD structures 3B0R, 4EMA, and 2Q59 bound to VCE-003.2 (yellow) with and without of GW9662 (orange). Binding of VCE-003.2 to the alternative binding site through Ser342 in the Ω loop β3 for crystals 4EMA (Ki = 2.11 μM), 2Q59 (Ki = 2.24 μM), and 3B0R (358.38 nM). In the presence of GW9662, VCE-003.2 binds to G284 of Helix 3 and Ser342 on the Ω loop and GW9662 binds to the canonical LPB site (Ki for VCE-003.2 = 711.27 nM). Analysis of VCE-003.2 binding to the canonical LBP site by interacting with Ser289 (2Q59: Ki for VCE-003.2 = 1.49 μM), R288 (3B0R: Ki for VCE-003.2 = 3.21 μM), and H449 (4EMA, Ki for VCE-003.2 = 607.81 nM) on Helix 11

To confirm the ability of VCE-003.2 to adopt different binding modes, we performed docking simulation studies using crystal structures 3B0R, 4EMA, and 2Q59 deposited in the Protein Data Bank (PDB). Molecular docking demonstrated that VCE-03.2 molecule binds to the hydrophobic core within the 11-helix bundle (canonical site) and also to the side of the Ω loop β3-β4 region in Helix 3 (alternative binding site). Moreover, in the presence of GW9662, VCE-003.2 only binds to the alternative site by interacting with Ser342 in the Ω loop β3 and Gly284 in Helix 3 (Fig. 9).

## Discussion

Whether neuroinflammation and microglia activation is a consequence or a cause of nigral cell deterioration and death in PD has been a controversial issue for years. However, it appears that presently, there is a general consensus about the important pathogenic role played by glial activation in this disease [39], with increasing evidence supporting that glia-driven inflammation contributes to the progressive degeneration of nigral dopaminergic neurons even in early phases of PD in patients [40]. Such situation may be experimentally reproduced using LPS insults into the nigrostriatal area [24], but it can be also found in other experimental models of PD [41] and identified in postmortem PD brains at autopsy [42]. In addition, epidemiological data support that the regular use of non-steroidal anti-inflammatory drugs (e.g., ibuprofen) reduces the risk of developing PD [43], a fact that gives an instrumental value to the control of inflammation in PD. Based on this idea, different anti-inflammatory agents have been investigated, at preclinical and even clinical levels, with variable success in PD. This includes inhibitors of iNOS or COX-2, non-steroidal anti-inflammatory drugs, the antibiotic minocycline, piaglitazone and other PPARγ activators, purinergic P2X receptor antagonists, and immunosuppressants [39, 42].

Cannabinoids have been also investigated for the reduction of inflammatory events in PD using mainly selective CB_2_ receptor agonists [2, 44]. The rationale to do that is based on the frequent upregulatory responses elicited by these receptors, predominantly in microglial cells and infiltrated macrophages recruited at the CNS areas lesioned in PD [4, 6], as well as on the anti-inflammatory and neuroprotective effects derived from the selective activation of these upregulated receptors [4, 6, 7, 9]. However, another line of evidence may derive from the possibility to target PPARγ receptors with certain cannabinoids in PD. Such evidence is based on (i) the relevant role played by these nuclear receptors in the control of inflammation in numerous pathological conditions [45, 46]; (ii) the well-described PPARγ-mediated anti-inflammatory activity of certain cannabinoids in different models of central and peripheral inflammation [11]; and (iii) the effects of non-cannabinoid PPARγ activators (e.g., thiazolidinediones [14]) in PD. PPAR receptors function as lipid sensors forming heterodimers with the retinoic acid X receptor and binding to regulatory DNA elements so-called peroxisome proliferator response elements (PPREs), then regulating transcription of a wide range of genes [47]. The three different PPAR isotypes (-α, -β/δ, and -γ) are expressed in all cell types in the brain [48, 49], and all of them, by functioning in an integrated manner as a complex system, so-called the PPAR triad, have been reported to have neuroprotective properties (reviewed recently in [47]).

In the present study, we attempted to activate PPARγ receptors using a CBG derivative, VCE-003.2, which has been previously found to be partial agonist at these receptors, and to investigate its potential in experimental models of PD. Thus, VCE-003.2 was investigated in a classic inflammatory rodent model of PD generated by intrastriatal application of LPS. This insult enhances reactive microgliosis, reflected by elevated Iba-1 and Cd68 immunostaining in the substantia nigra, in parallel to an elevation in the expression of proinflammatory markers in the striatum, including the cytokines TNF-α and IL-1β, and, in particular, iNOS. This is a pro-inflammatory enzyme which has been found to strongly contribute to deteriorate neuronal homeostasis in PD in conditions of reactive gliosis [50–52], a fact reflected in the loss of TH-positive neurons found here. We previously reported that the same proinflammatory markers were strongly elevated by the LPS insult and such elevations were partially reversed by the selective activation of CB_2_ receptors [6]. Using VCE-003.2, which does not activate CB_2_ receptors, we found the same response, reduction in the microglial reactivity in the substantia nigra and a marked reduction in the toxicity generated by these activated glial cells, which is reflected in the low levels of proinflammatory markers measured in the striatum of those LPS-lesioned animals treated with VCE-003.2. Such beneficial effects influenced the loss of TH-positive neurons in the substantia nigra, which was partially reduced. Our study also confirmed the involvement of an activation of PPARγ signaling in VCE-003.2 effects in LPS-lesioned mice, so in conditions of elevated glial reactivity and local inflammatory events. Such conclusion derives from the observation that the improvements reached with this CBG derivative in reactive microgliosis and the expression of proinflammatory markers disappeared when PPARγ receptors were blocked with a selective inhibitor that interacts with the canonical PPARγ LBP. Even, in some cases, the levels of reactive microgliosis and proinflammatory events appeared to be much higher after the blockade of PPARγ receptors, thus indicating the possible existence of an intrinsic endogenous activity of these nuclear receptors limiting inflammatory events that would disappear after the blockade with T0070907. A similar response was found in the study with VCE-003.2 conducted in experimental models of Huntington’s disease [19]. In this sense, it has been shown that endogenous fatty acid ligands such as 15d-PGJ_2_ and 5-oxo-ETE covalently bind to Cys285 in the PPARγ LBP and its transcriptional activities can be inhibited by T0070907 [53]. Thus, it is conceivable that in brain inflammatory conditions, some eicosanoids are produced to limit neuronal damage, and therefore, blocking this protective response would result in a more exacerbated damage.

Our in vivo study in LPS-lesioned mice was followed by investigation of the anti-inflammatory and neuroprotective properties of VCE-003.2 in a couple of in vitro experimental approaches using cultured LPS-exposed BV2 cells, alone or in combination with M-213 neuronal cells. Our objective was to confirm and, eventually, further explore in vitro the VCE-003.2-induced reduction in the LPS-induced generation of proinflammatory mediators, an objective that was completely attained with the first of the two in vitro approaches. Using only these microglia-related cells, we observed the expected increase in TNF-α, IL-1β, COX-2, and iNOS elicited by persistent exposure to LPS, which has been previously reported in other studies [54] and how VCE-003.2 was able, in general, to attenuate these responses. The second in vitro approach used in this study was aimed at confirming the capability of VCE-003.2 to protect neurons against glia-driven inflammatory insults. We generated conditioned media from cultured BV2 cells overexposed to LPS and added these media to cultured M-213 neuronal cells in combination with VCE-003.2 to determine its effects on cell viability. Again, VCE-003.2, used at low concentrations, was able to provide neuroprotection. We found that the in vitro anti-inflammatory, neuroprotective, and transcriptional (see below) activities of VCE-003.2 were not inhibited by either GW9662 or T0070907, which are PPARγ antagonists commonly used to discriminate between PPARγ-dependent and PPARγ-independent activities. However, this assumption is no longer valid, as there is evidence of alternative binding sites in the PPARγ LBP that are functional and not affected by this class of PPARγ antagonists. Indeed, our docking and functional assays strongly suggest that VCE-003.2 is a PPARγ partial agonist that mediates biological functions but acting through canonical and alternative PPARγ pathways. Thus, it is not surprising to find that the blockade of PPARγ with either GW9662 or T0070907 did not prevent some of the effects of VCE-003.2, even they were potentiated in some cases. Accordingly, it has been shown that T0070907 enhances the ability of some partial PPARγ agonists such as BVT.13 and nTZDpa to bind the PPARγ coactivator TRAP220 [20]. Equivalent data have been found for other biological activities [36, 37], as has been indicated in the “Results” section.

There is experimental evidence in the literature indicating that persistent LPS exposure in BV2 cells causes a downregulation in the expression of PPARγ receptors, which has been proposed to be elicited by elevated TNF-α synthesis [34, 35], as well as by an expected upregulation of NFκB signaling [55]. We found that VCE-003.2, as well as RGZ, induces the expression of PPARγ at the protein level and prevented LPS-induced downregulation of this protein. Since PPARγ gene expression is also upregulated by PPARγ ligands, our results further support a role for PPARγ as a major pathway activated by VCE-003.2. Nevertheless, we cannot exclude that VCE-003.2 induces PPARγ-independent effects and preliminary results indicates that this CBG derivative is also a dual inhibitor of the FAAH and MAGL activity (unpublished data) and is also an antioxidant (see Additional file 3). Thus, further research is warranted to investigate the mechanism of action of VCE-003.2 in neuroinflammatory conditions and to fully understand the differences between the in vivo (LPS-lesioned mice) and in vitro results regarding blockade by classic PPARγ antagonists. A possible explanation may be based on the differences between in vivo (complete cytoarchitecture) and in vitro (isolated cell populations) models. In this sense, it has been shown that serotonin metabolites, an important neurotransmitter with a potential relevant role in PD, binds and activates PPARγ [56], and it will be of interest to analyze the potential synergistic or additive activity of serotonin and VCE-003.2 on the PPARγ pathway. If the serotonin tone is lowered in PD patients, it will be possible to increase this tone in the presence of VCE-003.2, at least acting on PPARγ signaling.

Lastly, our study also addressed an additional objective as investigating the neuroprotective potential of VCE-003.2 dependent on its antioxidant profile in an experimental model of PD having poor microglial reactivity (see Additional file 3). We used 6-hydroxydopamine-lesioned mice, in which, as mentioned above, several antioxidant phytocannabinoids, such as cannabidiol, Δ^9^-tetrahydrocannabinol and Δ^9^-tetrahydrocannabidivarin, had been able to preserve TH-positive neurons in the substantia nigra through effects dependent on their cannabinoid receptor-independent antioxidant properties [7, 8, 21]. Our data indicated that this phytocannabinoid derivative was also beneficial in 6-hydroxydopamine-lesioned mice. However, contrarily, to the model of LPS lesions, its effects were more evident at the behavioral level (by improving motor impairment) than at the histopathological level. In fact, we were unable to detect a relevant effect on the preservation of TH-positive neurons and we found the expected lack of glial reactivity (see Additional file 3). We assume that these effects may reflect a certain protective effect based on the antioxidant activity of VCE.003-2, but it is possible that they simply reflect a mere symptom-relieving action not associated with preservation of nigral neurons.

## Conclusion

In summary, we have demonstrated that VCE-003.2 is neuroprotective against inflammation-driven neuronal damage in an in vivo model of PD, acting through the activation of the canonic binding site in PPARγ receptors. In vitro cellular models reminiscent of the in vivo PD pathology, however, indicated that the effects of VCE-003.2 to limit inflammation may be also exerted through additional mechanisms, including the possibility that VCE-003.2 targets PPARγ by acting through two binding sites at the LBP, one that is sensitive to T0070907 (canonical binding site) and other that is not affected by this PPARγ antagonist (alternative binding site). Collectively, our data support the idea of VCE-003.2 as a useful neuroprotective agent in PD based on its multi-target properties.

## Additional files

Additional file 1: Figure S1. Double immunofluorescence analysis of Cd68 (in green) and TH (in red) in the substantia nigra pars compacta of adult male mice at 3 weeks of being subjected to an intrastriatal injection of LPS. Top panels show both immunostainings in the contralateral (lesioned) and ipsilateral (non-lesioned) sides (scale bar = 50 μm), whereas bottom panels show the immunostaining for both markers and the merged image in the contralateral (lesioned) side (scale bar = 50 μm), proving the presence of Cd68 immunostaining associated with TH-positive cells, but a complete lack of overlapping, then demonstrating that they correspond to different cells (TH-positive neurons versus Cd68-positive microglial cells) (PDF 757 kb)

Additional file 2: Figure S2. BV2 cells were seeded at 1 × 10^5^ in 60-mm dishes and 24 h later treated with RGZ or VCE-003.2 in the absence or the presence of LPS (50 ng/mL) for 6 h and the steady state levels of endogenous PPARγ and β-actin detected by western blot (PDF 105 kb)

Additional file 3: Supplementary information and Supplementary Figures S3–S5. Effects of VCE-003.2 in 6-hydroxydopamine-lesioned mice (PDF 564 kb)

## Abbreviations

PPARγ: Peroxisome proliferator-activated receptor-γ
PPARγ2: PPARγ isoform 2
ADIPOQ: Adiponectin
CB_1_: Cannabinoid receptor type-1
CB_2_: Cannabinoid receptor type-2
CBG: Cannabigerol
CEBPA: CCAAT/enhancer-binding protein-α
COX-2: Cyclooxygenase-2
DMEM: Dulbecco’s modified Eagle’s medium
FABP4: Fatty acid-binding protein 4
FBS: Fetal bovine serum
IL-1β: Interleukin-1β
iNOS: Inducible nitric oxide synthase
KPBS: Potassium PBS
LBP: Ligand-binding pocket
LPL: Lipoprotein lipase
LPS: Lipopolysaccharide
MSCs: Mesenchymal stem cells
PBS: Phosphate-buffered saline
PD: Parkinson’s disease
PPREs: Peroxisome proliferator response elements
RGZ: Rosiglitazone
TH: Tyrosine hydroxylase
TNF-α: Tumor necrosis factor-α
