# Supplementary figures and images for: Benefits of VCE-003.2, a cannabigerol quinone derivative, against inflammation-driven neuronal deterioration in experimental Parkinson’s disease: possible involvement of different binding sites at the PPARγ receptor
Source: J Neuroinflammation. 2018 Jan 16;15:19. doi: 10.1186/s12974-018-1060-5 (PMC5771072; doi:10.1186/s12974-018-1060-5)

**Ipsilateral side (lesioned)**

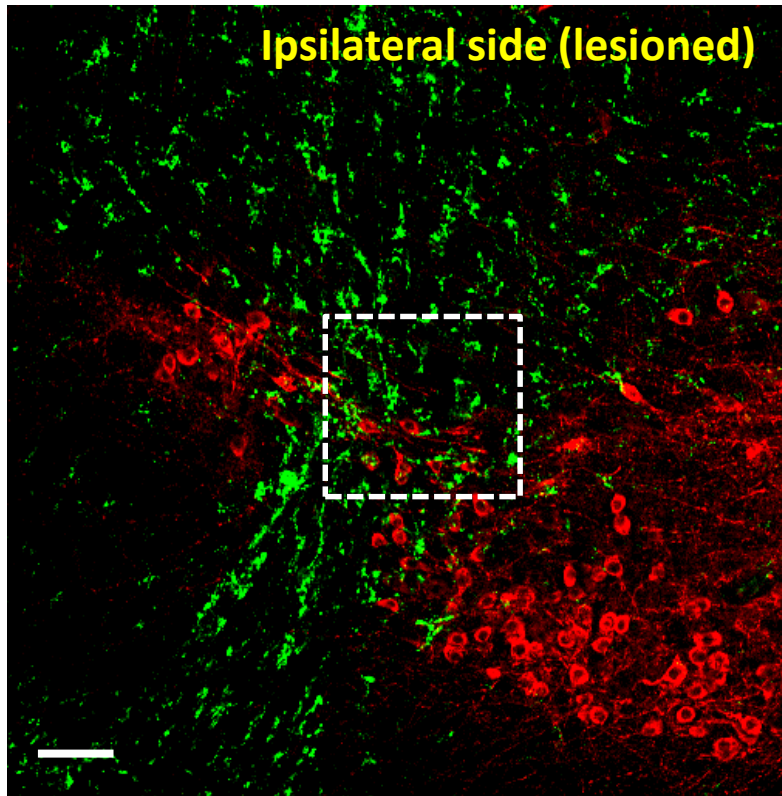

**Contralateral side (non-lesioned)**

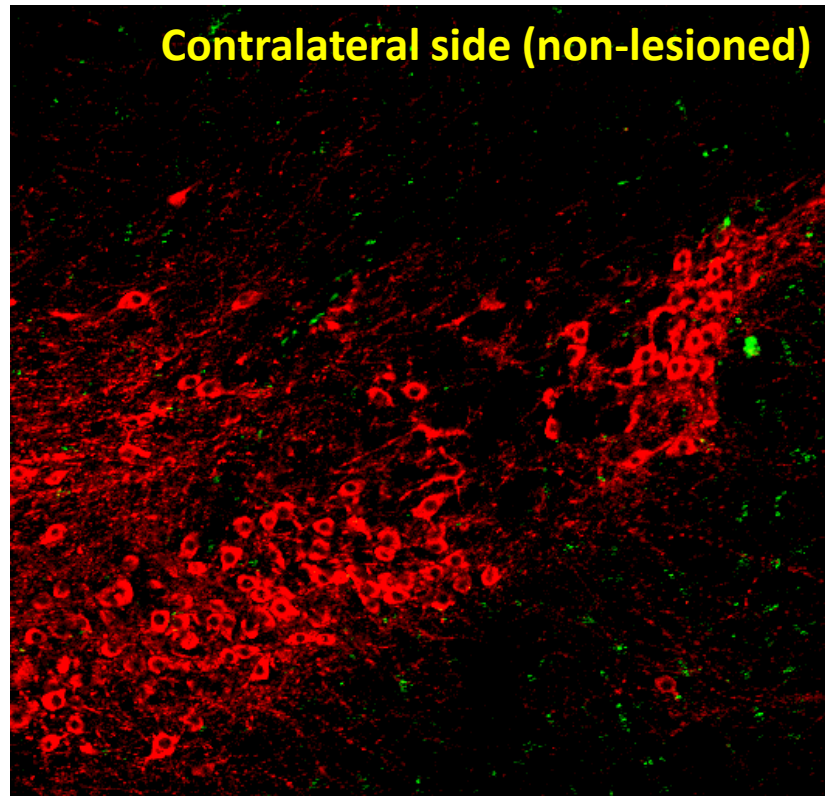

**Cd68**

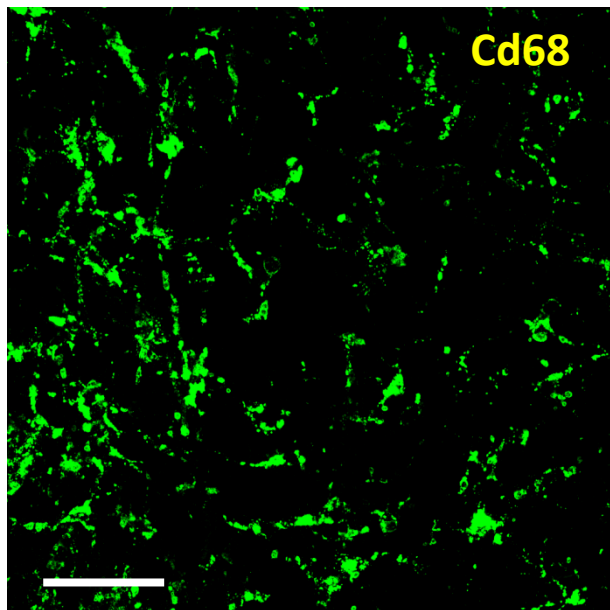

**TH**

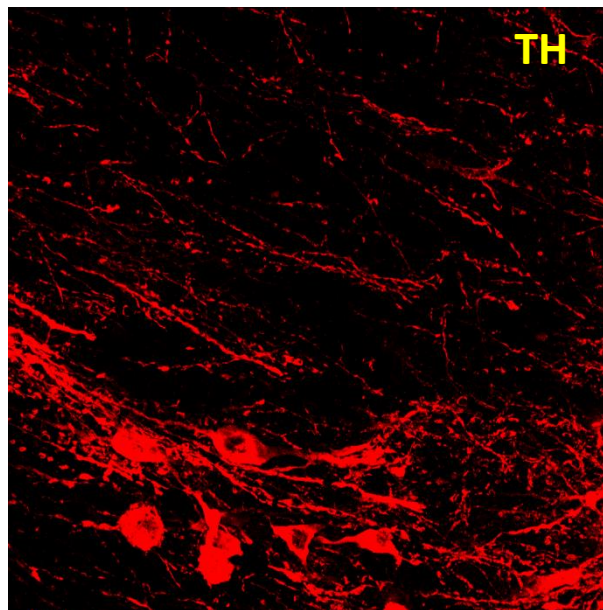

**merge**

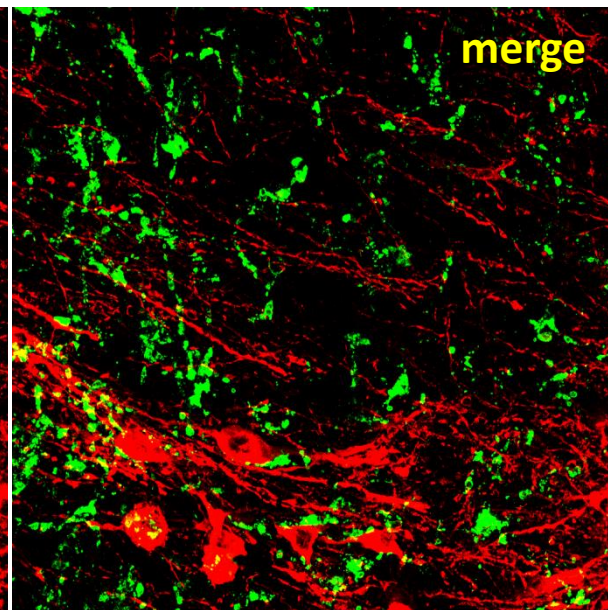

Supplement: Supplementary file 1 — Double immunofluorescence analysis of Cd68 (in green) and TH (in red) in the substantia nigra pars compacta of adult male mice at 3 weeks of being subjected to an intrastriatal injection of LPS. Top panels show both immunostainings in the contralateral (lesioned) and ipsilateral (non-lesioned) sides (scale bar = 50 μm), whereas bottom panels show the immunostaining for both markers and the merged image in the contralateral (lesioned) side (scale bar = 50 μm), proving the presence of Cd68 immunostaining associated with TH-positive cells, but a complete lack of overlapping, then demonstrating that they correspond to different cells (TH-positive neurons versus Cd68-positive microglial cells) (PDF 757 kb) [file 12974_2018_1060_MOESM1_ESM.pdf]

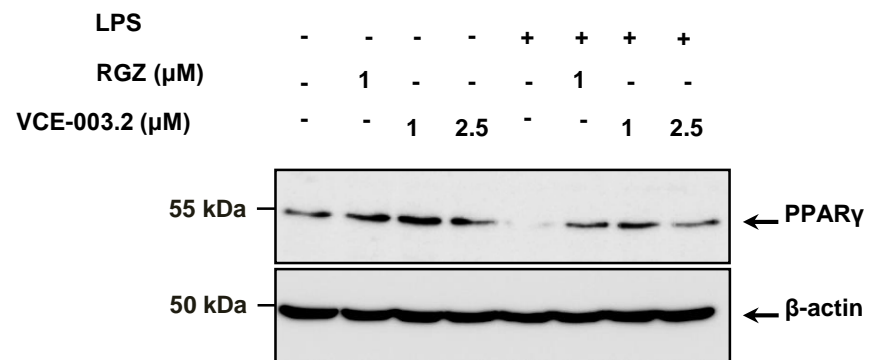

Supplement: Supplementary file 2 — BV2 cells were seeded at 1 × 10^5^ in 60-mm dishes and 24 h later treated with RGZ or VCE-003.2 in the absence or the presence of LPS (50 ng/mL) for 6 h and the steady state levels of endogenous PPARγ and β-actin detected by western blot (PDF 105 kb) [file 12974_2018_1060_MOESM2_ESM.pdf]
