# Supplementary material for: Benefits of VCE-003.2, a cannabigerol quinone derivative, against inflammation-driven neuronal deterioration in experimental Parkinson’s disease: possible involvement of different binding sites at the PPARγ receptor
Source: J Neuroinflammation. 2018 Jan 16;15:19. doi: 10.1186/s12974-018-1060-5 (PMC5771072; doi:10.1186/s12974-018-1060-5)

Benefits of VCE-003.2, a cannabigerol quinone derivative, against inflammation-driven neuronal deterioration in experimental Parkinson's disease: possible involvement of different binding-sites at the PPARγ receptor

Concepción García[1-3], María Gómez-Cañas[1-3], Sonia Burgaz[1-3], Belén Palomares[4-6], Yolanda Gómez-Gálvez[1], Cristina Palomo-Garo[1-3], Sara Campo[1], Joel Ferrer-Hernández[1], Carolina Pavicic[7], Carmen Navarrete[8], M. Luz Bellido[8], Moisés García-Arencibia[1-3], M. Ruth Pazos[1-3], Eduardo Muñoz[4-6] and Javier Fernández-Ruiz[1-3]

[1]Instituto Universitario de Investigación en Neuroquímica, Departamento de Bioquímica y Biología Molecular, Facultad de Medicina, Universidad Complutense, Madrid, Spain; [2]Centro de Investigación Biomédica en Red de Enfermedades Neurodegenerativas (CIBERNED), Madrid, Spain; [3]Instituto Ramón y Cajal de Investigación Sanitaria (IRYCIS), Madrid, Spain; [4]Instituto Maimónides de Investigación Biomédica de Córdoba (IMIBIC), Córdoba, Spain; [5]Departamento de Biología Celular, Fisiología e Inmunología, Universidad de Córdoba, Spain; [6]Hospital Universitario Reina Sofía, Córdoba, Spain; [7]Innohealth Group, Madrid, Spain; [8]Vivacell Biotechnology España, Córdoba, Spain.

Supplementary data

In an additional experiment presented as supplementary material, male C57BL/6 mice were anaesthesized (ketamine 40 mg/kg + xylazine 4 mg/kg, i.p.) 30 min after pretreatment with desipramine (25 mg/kg, i.p.), and then 6-hydroxydopamine free base (2 μl at a concentration of 2 μg/μl saline in 0.2% ascorbate to avoid oxidation) or saline (for control mice) were injected stereotaxically into the right striatum at a rate of 0.5 μl/min, using the following coordinates: +0.4 mm AP, ±1.8 mm ML and −3.5 mm DV, as described in Alvarez-Fischer and coworkers [1]. Once injected, the needle was left in place for 5 min before being slowly withdrawn. This avoids generating reflux and a rapid increase in intracranial pressure. Control animals were sham-operated and injected with 2 μl of saline using the same coordinates.

After the application of 6-hydroxydopamine, animals were treated with VCE-003.2 (10 mg/kg) administered i.p. for a period of 7 days (one injection *per* day) starting shortly (approximately 16 hours) after the lesion. Separate groups of sham-operated and 6-hydroxydopamine-lesioned animals received vehicle (DMSO-Tween 20-saline, 1.5-1-32). One day after the last injection, all animals were analysed in the rotarod test and in a computer-aided actimeter, at the end of which animals were transcardially perfused with saline followed by fresh 4% paraformaldehyde [in 0.1M phosphate buffered-saline (PBS)], and their brains were collected and postfixed overnight at 4ºC, cryoprotected with 30% sucrose in PBS and then frozen and stored at -80ºC for immunohistochemical analysis of the substantia nigra.

For the analysis of rotarod performance, we used a LE8200 device (Panlab, Barcelona, Spain). After a period of acclimation and training (first session: 0 rpm for 10s; second and third sessions: 4 rpm for 10s), animals were tested with an acceleration from 4 to 40 rpm over a period of 300s. Mice were tested for 3 consecutive trials and the mean of the 3 trials was calculated. For the analysis of motor activity, we used a computer-aided actimeter (Actitrack, Panlab, Barcelona, Spain). This apparatus consisted of a 45 x 45 cm area, with infra-red beams all around, spaced 2.5 cm, coupled to a computerized control unit that analyzes the following parameters: (i) distance run in the actimeter (ambulation); (ii) time spent in inactivity; (iii) frequency of vertical activity (rearing); (iv) mean and maximal velocity developed during the running; and (v) time spent in fast (> 5 cm/s) and slow (< 5 cm/s) movements. Animals remained for a period of 10 min in the actimeter, but measurements were only recorded during the final 5 min (first 5 min was used only for animal acclimation).

As mentioned in the main text, we wanted to investigate whether VCE-003.2 was active in a model of PD characterized by oxidative stress but poor glial reactivity/inflammatory events, as the one generated by unilateral lesions with 6-hydroxydopamine in mice, a model in which antioxidant phytocannabinoids were active by mechanisms independent of cannabinoid receptors [2-4]. Therefore, it is likely that the phytocannabinoid-based structure of VCE-003.2 enables this CBG derivative to be also active in this model. We found that VCE-003.2 significantly improved the motor impairment observed in 6-hydroxydopamine-lesioned mice. This

appeared as a mere trend in the rotarod test (see Supplementary Figure 3), but it was strongly evident in different motor parameters, such as the ambulatory activity ($F(2,12)=6.90$, $p<0.05$), resting time ($F(2,12)=8.78$, $p<0.01$), rearing behavior ($F(2,12)=10.74$, $p<0.005$), mean velocity ($F(2,12)=6.93$, $p<0.05$) and fast movement ($F(2,12)=5.35$, $p<0.05$), all measured in a computer-aided actimeter (see Supplementary Figure 3). However, the histopathological analysis of the substantia nigra of these mice showed only a very modest attenuation in the loss of TH-positive neurons in the substantia nigra ($F(2,14)=11.23$, $p<0.005$) (see Supplementary Figure 4), which only consisted in a reduction in the probability level compared to control animals. There was no changes neither by the lesion nor by the treatment with VCE-003.2 in glial markers Iba-1 ($F(2,14)=0.92$, ns) and Cd68 ($F(2,13)=2.43$, $P=0.134$) (see Supplementary Figure 4) measured by immunostaining, which is concordant with the poor inflammatory response typical of this experimental model.

We assume that the effects of VCE-003.2 in 6-hydroxydopamine-lesioned mice may be related to a potential antioxidant activity of this CBG derivative, a fact that has been investigated here using a procedure of detection of the intracellular accumulation of ROS by fluorescence using 2’,7’´dichlorofluorescein-diacetate (DCFH-DA). As depicted in Supplementary Figure 5, both CBG and VCE-003.2 showed potent antioxidant activity.

References

1. Alvarez-Fischer D, Henze C, Strenzke C, Westrich J, Ferger B, Höglinger GU et al. Characterization of the striatal 6-OHDA model of Parkinson's disease in wild type and alpha-synuclein-deleted mice. Exp Neurol 2008; 210: 182-193.
2. García C, Palomo-Garo C, García-Arencibia M, Ramos J, Pertwee R, Fernández-Ruiz J. Symptom-relieving and neuroprotective effects of the phytocannabinoid Δ^9-THCV in animal models of Parkinson's disease. Br J Pharmacol 2011; 163: 1495-1506.
3. García-Arencibia M, González S, de Lago E, Ramos JA, Mechoulam R, Fernández-Ruiz J. Evaluation of the neuroprotective effect of cannabinoids in

a rat model of Parkinson’s disease: importance of antioxidant and cannabinoid receptor-independent properties. Brain Res 2007; 1134: 162-170.

4. Lastres-Becker I, Molina-Holgado F, Ramos JA, Mechoulam R, Fernández-Ruiz J. Cannabinoids provide neuroprotection against 6-hydroxydopamine toxicity in vivo and in vitro: relevance to Parkinson's disease. Neurobiol Dis 2005; 19: 96-107.

Supplementary Figure 3

Rotarod performance and different behavioral responses measured in a computer-aided actimeter in adult male mice at 1 week of being subjected to an intrastriatal injection of 6-hydroxydopamine and daily treated with VCE-003.2 (10 mg/kg) or vehicle. Values are expressed as means ± SEM (n=5 subjects *per* group). Data were assessed by the one-way analysis of variance followed by the Student-Newman-Keuls test (*p<0.05, **p<0.01 *versus* vehicle-treated control mice; #p<0.05, ##p<0.01 *versus* vehicle-treated 6-hydroxydopamine-lesioned mice).

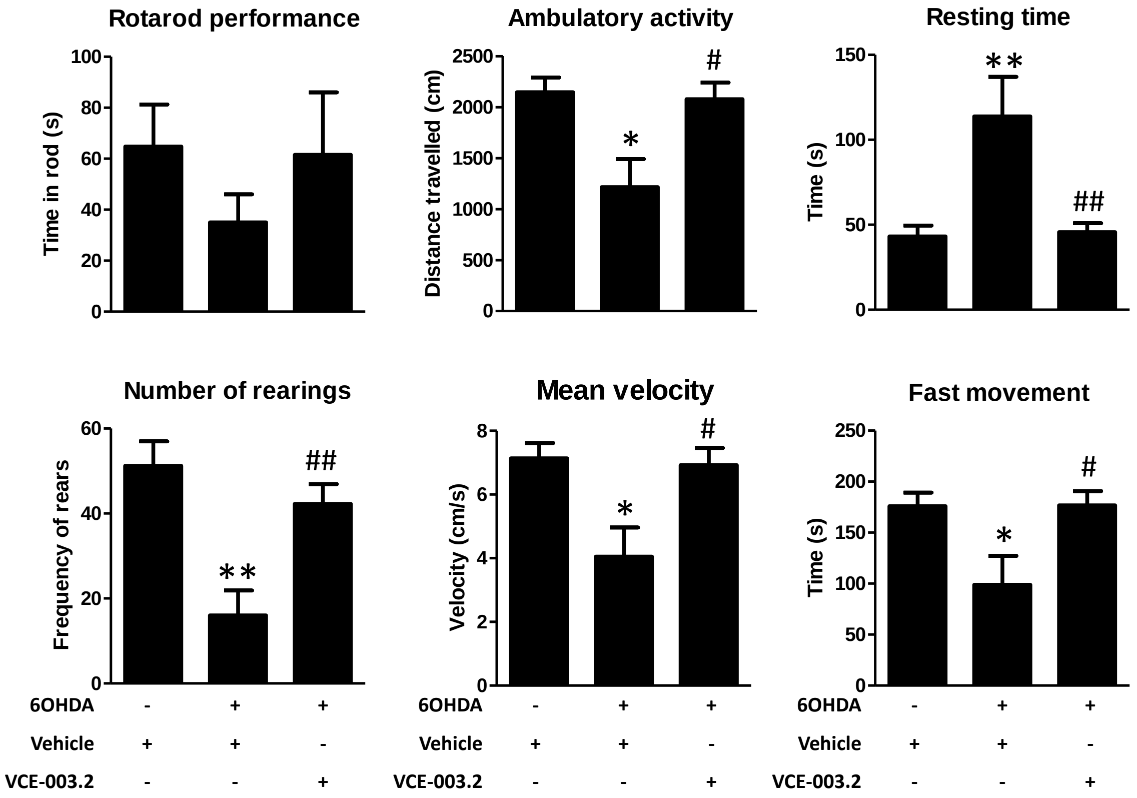

Supplementary Figure 4

Quantification of TH, Iba-1 and Cd68 immunoreactivity in the substantia nigra of adult male mice at 1 week of being subjected to an intrastriatal injection of 6-hydroxydopamine and daily treated with VCE-003.2 (10 mg/kg) or vehicle. Immunostaining values correspond to immunoreactivity levels in the lesioned side over the non-lesioned side and are expressed as means ± SEM (n=5 subjects *per* group). Data were assessed by the one-way analysis of variance followed by the Student-Newman-Keuls test (*p<0.05, **p<0.01 *versus* vehicle-treated control mice).

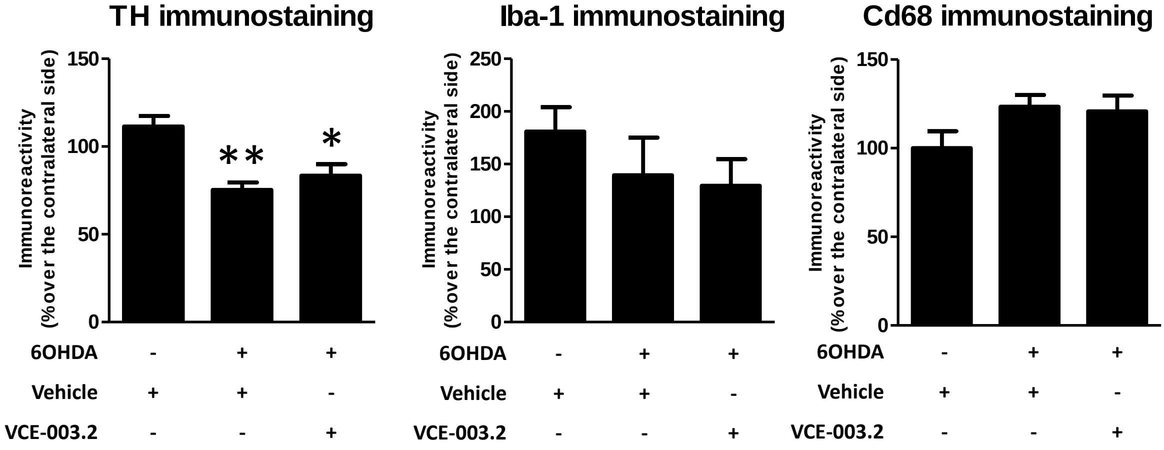

Supplementary Figure 5

SK-N-SH cells ($1x10^4$ cells/well) were cultured in a 96-well black plate in DMEM supplemented with 10% FBS; culture medium is renewed when the cells reached 80% confluence. For inhibition, the cells were pre-incubated with either CBG or VCE-003.2 at the indicated concentrations for 30 min and treated with 0,4 mM Tert-butyl-hydroperoxide (TBHP). After 3 h the cells were incubated with 10 μM DCFH-DA in the culture medium at 37ºC for 30 min. Then, the cells were washed with PBS at 37ºC and the production of ROS measured by changes in fluorescence due to the intracellular accumulation of DCF caused by the oxidation of DCFH. Intracellular ROS, as indicated by DCF fluorescence is detected using the Incucyte FLR software; the data are analyzed by the total green object integrated intensity ($GCUx\mu m^2xWell$) of the imaging system IncuCyte HD (Essen BioScience). Results are represented as the percentage of ROS^+ cells considering 100% for TBPH-treated cells.

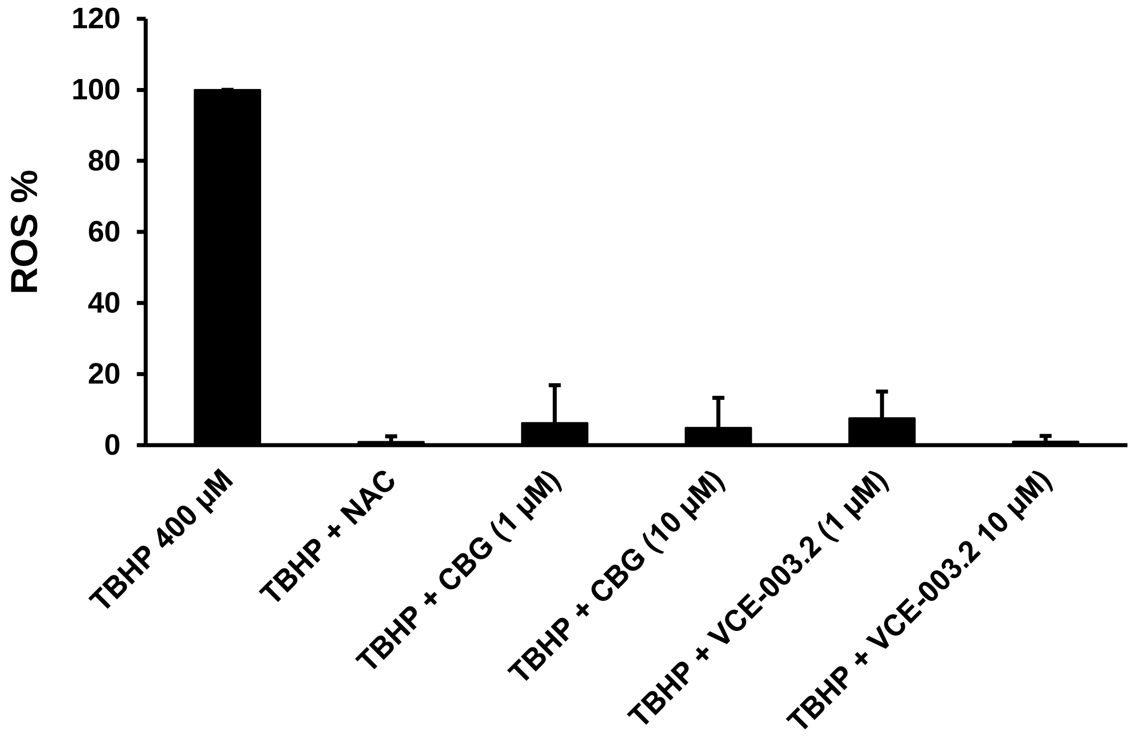

Supplement: Supplementary file 3 — Supplementary information and Supplementary Figures S3–S5. Effects of VCE-003.2 in 6-hydroxydopamine-lesioned mice (PDF 564 kb) [file 12974_2018_1060_MOESM3_ESM.pdf]
